# A novel scheme for ultrashort terahertz pulse generation over a gapless wide spectral range: Raman-resonance-enhanced four-wave mixing

**DOI:** 10.1038/s41377-023-01071-z

**Published:** 2023-02-02

**Authors:** Jiaming Le, Yudan Su, Chuanshan Tian, A. H. Kung, Y. Ron Shen

**Affiliations:** 1 Department of Physics, State Key Laboratory of Surface Physics and Key Laboratory of Micro- and Nano-Photonic Structure (MOE), Fudan University, Shanghai, 200433 China; 2 Department of Physics, University of California, Berkeley, CA 94720 USA; 3 Collaborative Innovation Center of Advanced Microstructures, Nanjing, 210093 China

**Keywords:** Nonlinear optics, Terahertz optics

## Abstract

Ultrashort energetic terahertz (THz) pulses have created an exciting new area of research on light interactions with matter. For material studies in small laboratories, widely tunable femtosecond THz pulses with peak field strength close to MV cm^−1^ are desired. Currently, they can be largely acquired by optical rectification and difference frequency generation in crystals without inversion symmetry. We describe in this paper a novel scheme of THz pulse generation with no frequency tuning gap based on Raman-resonance-enhanced four-wave mixing in centrosymmetric media, particularly diamond. We show that we could generate highly stable, few-cycle pulses with near-Gaussian spatial and temporal profiles and carrier frequency tunable from 5 to >20 THz. They had a stable and controllable carrier-envelop phase and carried ~15 nJ energy per pulse at 10 THz (with a peak field strength of ~1 MV cm^−1^ at focus) from a 0.5-mm-thick diamond. The measured THz pulse characteristics agreed well with theoretical predictions. Other merits of the scheme are discussed, including the possibility of improving the THz output energy to a much higher level.

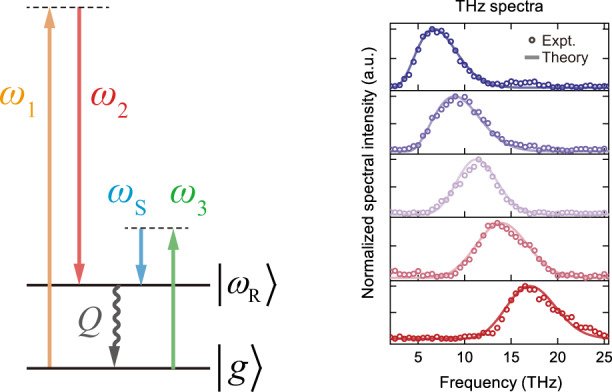

## Abstract (web summary)

Energetic ultrashort THz pulses tunable over a wide gapless spectral region can be generated by Raman-resonance-enhanced four-wave mixing in diamond.

## Introduction

The advent of intense ultrashort coherent light pulses has revolutionized the spectroscopy field^1–3^. On one hand, they allow easy observation of various nonlinear optical effects and facilitate characterization of materials with nonlinear optical spectroscopy. On the other hand, they provide means for strong selective excitation of materials, and optical manipulation of material structure and properties such as optical-field-induced ferroelectricity, superconductivity, and others^4–6^. They have also created the vibrant field of ultrafast dynamics of low-frequency excitations in materials^1^. Thanks to advances in laser technology over the past decades, intense femtosecond (fs) pulses from table-top setups are now available over essentially the whole spectral range from ~10 THz to soft X-ray even in ordinary laboratories. Below ~5 THz, high-intensity picosecond pulses exist^7^, but between 5 and 12 THz, stable, continuously tunable, energetic fs pulses are more difficult to come by^8–10^. Yet this is a spectral range of great importance for materials studies. Phonons and vibrations of molecules and solids composed of heavier atoms are in this range, and so are the intermolecular (or inter-molecular-group) vibrations of molecular systems in chemistry and biology. Various elementary excitations of solids also lie in this spectral region. In the review article on “The 2017 Terahertz Science and Technology Roadmap”, the 5 to 15 THz spectral range was labeled as the present-day THz gap calling to be filled^8,9^.

Currently, optical rectification (OR) or difference frequency generation (DFG) in second-order nonlinear crystals is the common technique to generate intense THz pulses. However, because of phonon absorption, THz generation from crystals is limited. For example, GaSe is among the few best nonlinear crystals for THz generation^11–13^, but it has several absorption bands between 10 and 20 THz and a precipitous drop in transmission below 10 THz^14,15^. The strong dispersion of the reststrahlen band at ~8 THz limits the effective coherent length of fs DFG in GaSe to ~200 μm in the 10–20 THz range (with pump wavelength around 1 µm) and distorts the THz temporal profile. The effective coherence length becomes much less than 100 μm below 10 THz. Organic nonlinear crystals have been used for efficient THz generation, but they have only a few narrow transparent windows above 5 THz and suffer from low optical damage thresholds^16^. Laser-induced gas plasmas can generate energetic THz pulses with spectra peaked at low THz and a broad wing extending to >50 THz. They are well suited as probes for linear THz spectroscopy over a wide spectral range, but their complex spatial mode patterns make them less desired as pumps for strong resonant excitations^17–19^. Free electron lasers and electron-driven THz sources can also produce fs pulses covering the entire THz spectral range^20,21^, but they are not readily available for most researchers. One may think four-wave mixing as a frequency conversion process could generate desirable THz pulses from materials if the input beams are sufficiently intense.

Indeed, THz generations by four-wave mixing^22^ and seeded optical Kerr amplification^23^ in crystals were proposed earlier, but they ran into difficulties in practice. The third-order nonlinear susceptibilities of materials are small (*χ*^(3)^ ~ 10^−14^ to 10^−15^ esu or 10^−22^ to 10^−23^ m^2^ V^−2^). Generation of >10 nJ THz pulses requires input pulses with peak intensities higher than a few TW cm^−2^, which is above the optical damage threshold of most materials. The scheme can be made practical only if *χ*^(3)^ of a material can be greatly enhanced through resonances while the optical damage threshold is still sufficiently high. This is achievable in wide band gap materials with two of the inputs exciting a Raman resonance. Of these materials, diamond is most attractive because of its unique absorption spectrum (see Supplementary Information, SI, for more details).

Diamond has no intrinsic absorption throughout the spectral range from 0 to 5.5 eV (44,000 cm^−1^) except for some minor absorption between 1600 and 4000 cm^−1^ due to multi-phonon transitions. It possesses a nonresonant *χ*^(3)^ of ~4.6 × 10^−14^ esu (6.4 × 10^−22^ m^2^ V^−2^) that is resonantly enhanced to ~6 × 10^−12^ esu (8 × 10^−20^ m^2^ V^−2^) near the Raman resonance at 40 THz^24^. The optical damage threshold of a typical CVD diamond plate is ~7 TW cm^−2^ for ~60 fs pulses at 50 THz in the infrared (measured value on our CVD diamond at 50 THz). With all three inputs in the infrared, noncollinear phase matching in four-wave mixing is possible from 2.5 to >100 THz. We report here a successful demonstration of THz generation by resonant four-wave mixing (R-FWM) in diamond. We were able to generate stable, few-cycle, transform-limited THz pulses that were tunable from 5 to >20 THz with controllable carrier-envelope phase (CEP) and had ≥10 nJ per pulse. This THz generator nicely covers the existing THz gap in THz spectroscopy^8,9^. We summarize in Table 1 the data on THz pulse generation by different schemes commonly used in small laboratories for comparison.

**Table 1:** Summary of THz generation schemes commonly used in small laboratories

Scheme	Reference	Input parameters		Output characteristics			
		Wave-length (μm)	Pulse energy (μJ)	Peak freq. (THz)	Bandwidth (THz)	Pulse energy (μJ)	Conversion (‰)
OR in LiNbO_3_	Ref. ^25^	0.8	6.5 × 10^3^	0.35	0.5	6.4	1
Gas plasma	Ref. ^26^	0.8	560	6	12	~0.06^a^	~0.1^a^
DFG in GaSe	Ref. ^11^	1.1	~300	30	10	1.7	~6
		~1.2	~300				
DFG in GaSe	Ref. ^13^	1.03	~3	13	6	1.5 × 10^–3, b^	0.5^b^
		1.07	4.7				
R-FWM in diamond	This work	1.45	85	5	9	5 × 10^–3^	0.5
		1.80	35	10		1.5 × 10^–2^	1.5
		~6	10	15		4.1 × 10^–2^	4

^a^Estimated with 10^–4^ yield

^b^Scaled from THz generation at higher frequency in ref. ^13^. The pulse repetition rate was 190 kHz

### Theory for THz generation by Raman-resonant four-wave mixing in diamond

**Fig. 1:**
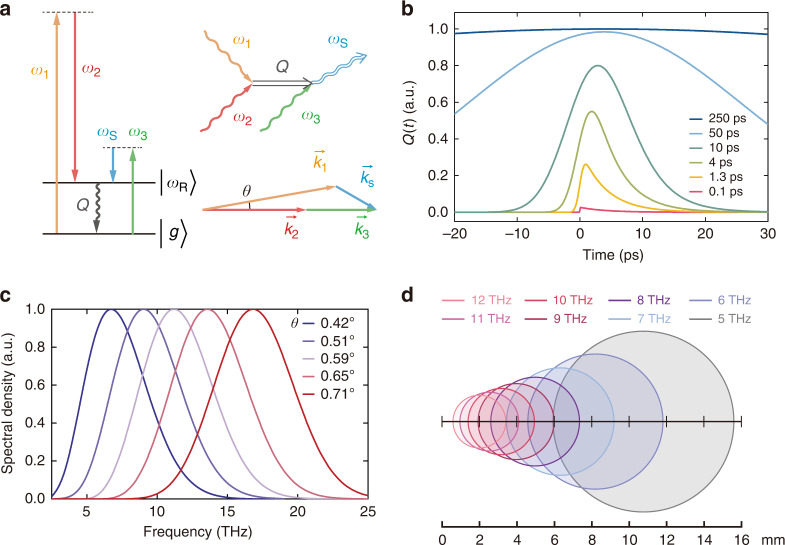
THz generation by R-FWM in diamond. **a** Energy level diagram, Feynman diagram, and wave-vector diagram describing four-wave mixing with a Raman resonance. **b** Time variation of the 1332 cm^−1^ vibrational wave in diamond coherently excited by $\vec E_1$ and $\vec E_2$ pulses of different pulse widths. **c** Calculated THz output spectra at selected *θ*. **d** Calculated spatial distributions of different frequency components in the collimated THz output beam for the case of *θ* = 0.42° in **c**; each circle describes a Gaussian distribution cut off at e^−2^ of the maximum

The basic theory behind resonant four-wave mixing (R-FWM) is straightforward. The three pulsed inputs, described by $\vec E_i\left( {\omega _i,\vec r,t} \right) = \vec E_{{{{\mathrm{ }}}}i}\left( {\vec r,t} \right)\exp ( {i\vec k_i \cdot \vec r - i\omega _it} )$ with center frequency $\omega _i$ (*i* = 1, 2, and 3), induce a third-order nonlinear polarization $\vec P^{\left( 3 \right)}\left( {\omega _{{{\mathrm{S}}}} = \omega _3 - \omega _1 + \omega _2,\vec r,t} \right)$ with center frequency at $\omega _{{{\mathrm{S}}}}$ in a medium, where $\omega _1 - \omega _2\sim \omega _{{{\mathrm{R}}}}$ with $\omega _{{{\mathrm{R}}}}$ being the Raman resonance frequency (Fig. 1a). Knowing $\vec P^{\left( 3 \right)}\left( {\omega _{{{\mathrm{S}}}},\vec r,t} \right)$, we can solve the wave equation to find the output field $\vec E_{{{\mathrm{S}}}}\left( {\omega _{{{\mathrm{S}}}},\vec r,t} \right)$. For generation of fs THz pulses, however, a few details should be noted (see SI for details). The Raman linewidth of diamond is ~1.25 cm^−1^, corresponding to a ~4 ps vibrational dephasing time. This means that fs input pulses of $\vec E_1$ and $\vec E_2$ can only excite the Raman resonance weakly. To fully benefit from the Raman resonance enhancement of *χ*^(3)^, we must employ picosecond $\vec E_1$ and $\vec E_2$ pulses for excitation.

The R-FWM process can be understood as follows^27^: The ps $\vec E_1$ and $\vec E_2$ input pulses coherently excite the transient vibrational wave, $\vec Q\left( {\vec r,t} \right)$, associated with the Raman resonance in diamond; the fs $\vec E_3$ input pulse then beats with the vibrational wave in diamond at a selected time to create the fs THz pulse. We can find $\vec Q\left( {\vec r,t} \right)$ from given $\vec E_1\left( {\vec r,t} \right)$ and $\vec E_2\left( {\vec r,t} \right)$ by solving the equation of motion for $\vec Q\left( {\vec r,t} \right)$. We can write $Q\left( t \right) \equiv f\left( t \right)Q_{{{{\mathrm{st}}}}}$, where $Q_{{{{\mathrm{st}}}}} \propto E_{1{{{\mathrm{m}}}}}E_{2{{{\mathrm{m}}}}}$ is the steady-state *Q*(*t*) that would appear if $E_1\left( t \right)$ and $E_2\left( t \right)$ with peak values of *E*_1m_ and *E*_2m_ were long pulses. Beating of $\vec E_3\left( {\vec r,t} \right)$ and $\vec Q\left( {\vec r,t} \right)$ then induces $\vec P^{(3)}\left( {\omega _{{{\mathrm{S}}}},\vec r,t} \right) = {\overleftrightarrow{\chi}}_{{\rm{res}}}^{(3)}\left( t \right):\vec E_{1{{{\mathrm{m}}}}}^ \ast \left( {\vec r} \right)\vec E_{{{{\mathrm{2m}}}}}\left( {\vec r} \right)\vec E_3\left( {\vec r,t} \right)$ with ${\overleftrightarrow{\chi}}_{{\rm{res}}}^{(3)}({\it{t}}) = {\it{f}}({\it{t}})( {{\overleftrightarrow{\chi}}_{{\rm{res}}}^{(3)}} )_{{{{\mathrm{st}}}}}$. Plotted in Fig. 1b is the calculated *Q*(*t*) for diamond under Gaussian pulse excitation of $\vec E_1^ \ast \left( t \right)\vec E_2\left( t \right)$ with different pulse widths. It is seen that for a pulse width of 1.3 ps, *Q*(*t*) can reach a peak value of ~30% of $Q_{{{{\mathrm{st}}}}}$ in diamond with a corresponding ${\overleftrightarrow{\chi}}_{{\rm{res}}}^{3}\left( t \right) \sim 0.3\left( {\overleftrightarrow{\chi}}_{{\rm{res}}}^{3} \right)_{{{{\mathrm{st}}}}}$.

With $\vec P^{\left( 3 \right)}\left( {\omega _{{{\mathrm{S}}}},\vec r,t} \right)$ known from given $\vec E_1\left( {\vec r,t} \right),\,\vec E_2\left( {\vec r,t} \right)\,{{{\mathrm{and}}}}\,\vec E_3\left( {\vec r,t} \right)$, we can find the THz output by solving the wave equation or by numerically calculating the output field from the dipole radiation equation^28^. Details of the calculations are presented in SI. For quantitative evaluation, we considered the case of 1.3 ps *E*_1_ and *E*_2_ pulses at 206 and 166 THz, respectively, to excite the Raman resonance of a 500-μm diamond (001) plate and a 60 fs *E*_3_ pulse with center frequency tunable from 45 to 60 THz to generate 5 to 20 THz output; all beams were p-polarized along [110] and incident on diamond at ~45°. The wave vector diagram sketched in Fig. 1a shows that noncollinear phase matching of the R-FWM process is possible. For each *θ* between $\vec k_1$ and $\vec k_2$||$\vec k_3$, there is a corresponding phase-matched (PM) THz output with a bandwidth of a few THz that satisfies the near-phase-matching condition of $\Delta kl/2 \,<\, 1$, where *l* is the beam path length in diamond^24^. Thus, the transform-limited THz output should appear as fs pulses. The calculated THz output spectra for different *θ* are described in Fig. 1c. Because of the $\omega _{{{\mathrm{S}}}}^2$ dependence of THz generation, the peaks of the spectra are blue shifted from those expected from PM. The near-PM requirement also leads to emission of different THz components in different directions with different angular spreads (see SI and Fig. S3). Collimation after the beam exit from diamond makes different THz components appear with differently displaced near-circular Gaussian distributions in the collimated beam. Figure 1d is an example showing the PM 5 THz distribution at the center and those of other frequency components away from the center. Focusing of the collimated beam or imaging of the beam exit from diamond recombined all the THz frequency components at the image position.

For a crude estimate on the THz output, we can use the usual expression for output energy per pulse from wave mixing under the pump-depletion-less limit. The energy per pulse for THz generation from phase-matched four wave mixing is given by:

$${{{\mathrm{ }}}}W\left( {\omega _{{{\mathrm{S}}}}} \right)\sim \frac{{16\pi ^4\omega _{{{\mathrm{S}}}}^2}}{{c^4n_1n_2n_3n_{{{\mathrm{S}}}}}}\left| {\chi ^{\left( 3 \right)}l} \right|^2\frac{{W\left( {\omega _1} \right)W\left( {\omega _2} \right)}}{{A^2T^2}}W\left( {\omega _3} \right)$$

in cgs units, where *A* is the beam overlapping area, *T* is the pulse width of $\vec E_1$ and $\vec E_2$, and *l* is the interacting path length. For the diamond case, if we take $|{\chi ^{(3)}} | = 0.3| {( {\chi _{{{{\mathrm{res}}}}}^{\left( 3 \right)}} )_{{{{\mathrm{st}}}}}}|\sim 2 \times 10^{ - 12}{{{\mathrm{ esu}}}}$ (~3 × 10^−20^ m^2^ V^−2^), *l* = 0.5 mm, *W*(*ω*_1_) = *W*(*ω*_2_) = 40 μJ, *W*(*ω*_3_) = 10 μJ, *A* = 4 × 10^−4^ cm^2^, and *T* = 1.3 ps, we find *W*(*ω*_s_) ~ 14 nJ per pulse at *ω*_s_/2π = 10 THz with a pulse width nearly the same as that of the $\vec E_3$ input. This suggests that R-FWM in diamond can be a powerful THz generator.

### Experimental arrangement

In the experiment, we used a commercial 33-fs, 5-W,1-kHz Ti:sapphire laser system to pump two optical parametric amplifiers (OPA) seeded by a common white light source. (Details of the experimental arrangement is given in SI). The signal and idler from OPA-1 centered at *ω*_1_/2π = 206 THz and *ω*_2_/2π = 166 THz were stretched to 1.3-ps positively chirped pulses with a constant instantaneous difference frequency of 40 THz. The *ω*_3_ pulses of 63-fs width, tunable between 40 and 60 THz were derived from OPA-2 with a difference frequency generation stage. All three p-polarized pulses were incident on a 0.5-mm (001)-cut diamond plate at ~45° and overlapped over an area inside diamond of π(140 × 100)/2 μm^2^. The p-polarized THz outputs were characterized by knife-edge beam profiling, a Fourier transform infrared interferometer (FTIR) and electro-optic sampling (EOS) (see SI for details).

## Results

**Fig. 2:**
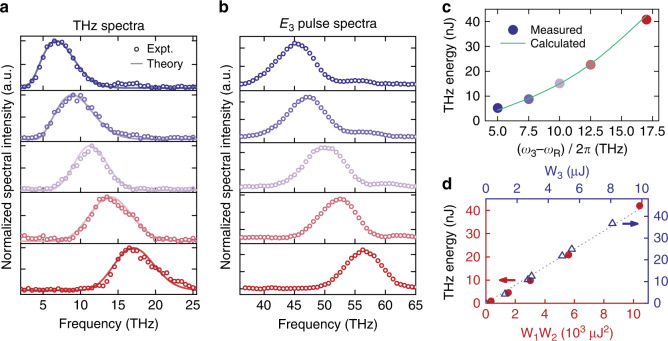
Experimental results on R-FWM in diamond. **a** THz output spectra: top to bottom frames for beam geometry set to have the THz frequency PM at 5, 7.5, 10, 12.5, and 17 THz, respectively. Solid curves are theoretical fits. **b** Measured one-to-one corresponding spectra of the converting $\vec E_3$ pulses with respect to the THz spectra in (**a**). **c** Output THz energy per pulse (circles) for the five beam geometries set in **a** and calculated THz energy (curve) using $\chi ^{(3)} = 2.8 \times 10^{ - 12}$esu. **d** Data showing the linear dependences of THz pulse energy on energy product of *ω*_1_ and *ω*_2_ pulses and on energy of the *ω*_3_ pulse

Figure 2a shows a set of measured THz spectra generated by R-FWM with the beam geometry set to have *θ* at 0.42°, 0.51°, 0.59°, 0.65°, and 0.71°. The input energies per pulse were *W*_1_ ≈ 85 μJ, *W*_2_ ≈ 35 μJ and *W*_3_ ≈ 10 μJ on the overlapping area. The corresponding spectra of the *ω*_3_ input pulses generating the THz spectra are given in Fig. 2b. It is seen that the two sets of spectra are very similar as expected except for an overall shift of 40 THz and some deviation due to the $\omega _{{{\mathrm{S}}}}^2$ dependence of THz generation. The measured THz spectra can be well fit by theory as described in Fig. 2a. The total THz energy versus the THz frequency $\omega _3 - \omega _{{{\mathrm{R}}}}$ is plotted in Fig. 2c. It is 5 nJ per pulse at 5 THz and increases to 41 nJ per pulse at 17 THz. In Fig. 2d, the output energy, *W*(*ω*_s_), is seen to be linearly proportional to the input energies, *W*_1_*W*_2_ and *W*_3_, as it should be. From the measured THz energies, we were able to deduce $| {( {\chi _{{{{\mathrm{res}}}}}^{( 3)}})_{{{{\mathrm{st}}}}}} |\sim 9 \times 10^{ - 12}\,{{{\mathrm{esu}}}}$ (~1 × 10^−19^ m^2^ V^−2^), compared to $6 \,\times \,10^{ - 12}\,{{{\mathrm{esu}}}}$ in ref. ^24^ We also used a slit of variable width placed at various positions in the output beam to map out the spatial distribution of the THz output spectrum and found good agreement with theoretical prediction (see SI and Fig. S6). As expected, the THz output was spatially chirped (see Fig. 1d). With a pair of parabolic mirrors, the THz output re-focused at the image point appeared as transformed-limited fs pulses (see SI and Fig. S7). An appropriate aperture in the THz beam path could select a specific portion of the THz output to yield a THz pulse with the desired spectral and temporal profiles.

**Fig. 3:**
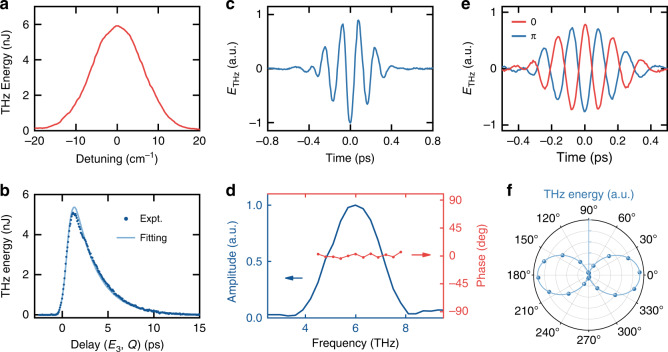
Characteristics of THz generation by R-FWM in diamond. **a** THz output energy versus resonance detuning. **b** Dependence of THz output energy with time relative to the vibration excitation *Q*(*t*) and its fit with |*Q*(*t*)|^2^. **c** Temporal trace of a THz pulse with PM at 5 THz measured by EOS in GaP. **d** Amplitude and phase spectra from Fourier transform of the THz pulse in (**c**). **e** THz pulses with CEP of 0 (red curve) and π (blue curve). **f** Measurement (scatters) and fitting (solid curve) of polarization distribution of 17 THz output showing that the THz pulses were linearly polarized

Figure 3a provides a description on the effect of detuning of excitation from Raman resonance. As $\omega _1 - \omega _2$ moves away from the resonance, the observed THz output drops as predicted. In another measurement, the overlapping time of the fs *E*_3_ pulse with the excited vibration, *Q*(*t*), was varied, the THz output went through a peak and then decayed, mapping out the variation of *Q*(*t*), as depicted in Fig. 3b. Theoretical fit of the data allowed the deduction of the phonon dephasing time, *T*_2_ = 4.8 ps.

We also characterized the THz output in the time domain by EOS. The result for a THz output pulse PM at 5 THz is displayed in Fig. 3c with its Fourier-transformed amplitude and phase spectra in Fig. 3d. Due to absorption at higher THz frequencies in the EOS crystal, the EOS temporal trace was distorted into a broader pulse and a corresponding narrower FT spectrum compared to that measured directly by FTIR. The observed uniform spectra phase in Fig. 3d however implies that the THz pulse should be nearly transform-limited with a pulse width of ~70 fs. If such a pulse is focused to an area of ~(100 μm)^2^, the peak electric field of 10 and 17 THz pulses with energy of 15 nJ and 41 nJ can reach about 1 and 1.7 MV cm^−1^, respectively. The THz pulses were CEP locked, and the phase could be controllably changed by varying the time delay of the *ω*_3_ pulse (see SI). Figure 3e shows two measured THz pulses with CEP adjusted to 0 and π; a time delay of 13 fs of the *ω*_3_ pulse led to a CEP change of π. The THz output generated was linearly polarized in the same plane as the inputs as described in Fig. 3f.

## Discussion

The above results constitute the first demonstration that R-FWM in centrosymmetric condensed media can be effective to generate energetic THz pulses. Diamond, in particular, has attractive merits. Its high optical damage threshold allows input pump intensities to be so high as to induce third-order nonlinear optical effects (e.g., FWM) competing with second-order effects (e.g., DFG) in other crystals. Transparency throughout the region from THz to UV leads to weak frequency dispersion of optical response coefficients that facilitates phase-matched THz pulse generation over a very wide spectral range with exceptionally good quality. With the phonon mode Raman-excited by ps pulses, the coherent phonon wave hardly changes on the fs time scale. It is then seen that R-FWM for fs THz pulse generation in diamond is effectively a beating process between a fs IR pulse with a constant phonon wave or a frequency conversion process down-converting the fs IR pulse by the phonon wave to the fs THz pulse. The latter essentially duplicates the input fs IR pulse except for a shift in frequency and some modification due to radiation efficiency. Thus, the characteristics of the THz pulse can be readily tuned by tuning the corresponding characteristics of the input IR pulse. The energy conversion efficiency is given directly by the ratio of the output THz pulse energy to the input fs pulse energy. We have measured phase-matched THz pulse generation with the center frequency spanning from 5 to 20 THz, but this spectral range can be readily extended to >100 THz. As the THz radiation efficiency increases with square of the frequency, and there is more pulse energy at higher input frequency, we expect the output energy at higher THz frequency generated by R-FWM in diamond can reach a level comparable to that generated by DFG in GaSe or other crystalline compounds.

As a first demonstration of THz generation by R-FWM, we have not optimized the parameters in our experiment. We can increase the peak intensities of the three input pulses on diamond by an overall factor of 10 before reaching the optical damage threshold. Doubling the diamond thickness would increase the conversion efficiency by ~4. If more input energy from the laser system is available to pump a larger area of diamond, the THz output energy can be further improved in proportional to the beam area. While the R-FWM scheme is more complex in experimental arrangement, it has obvious merits compared to the other schemes listed in Table 1, namely, the wide tunability of the center frequency and band width of the fs THz pulses, their near-Gaussian spatial and temporal profiles, and the relatively high conversion efficiency from IR input to THz output.

### Summary and prospects

Our study has unequivocally established that R-FWM in diamond can be made into a high-quality, powerful, fs THz generator over a wide spectral range without any gap. For fs THz pulse generation, R-FWM in diamond can be regarded as a direct frequency down-conversion process that converts a fs input pulse at higher frequency to a fs THz pulse that has characteristics essentially duplicating the characteristics of the input pulse except for the frequency shift. An input pulse of high quality, in terms of spatial, spectral, and temporal profiles as well as amplitude and phase stability, generates a THz pulse of nearly equal high quality. Tuning the characteristics of the input pulse tunes the characteristics of the THz output pulse accordingly. In this respect, we can amplitude- or polarization-modulate the THz pulse simply by modulating the input pulse^29^.

With a fs commercial laser system, we were able to use R-FWM in diamond to generate stable fs THz pulses over a wide spectral range with very high quality. The THz output energy was >10 nJ (producing a peak field strength ~1 MV cm^−1^ at a 100-μm focal spot) at 10 THz, and higher for higher THz frequencies. It could be enhanced by at least one order of magnitude with a higher input pulse energy and a thicker diamond plate. We also note that R-FWM in diamond can be collinearly phase-matched if the diamond plate is made birefringent by uniaxial stress of ~1 GPa. Growth of CVD diamond to >6 mm thick was recently reported^30^; collinear PM in such a diamond plate would push R-FWM toward the pump depletion region and a THz output of >1 μJ per pulse should be easily attainable with an input of ~10 μJ per pulse from the converting pulses, corresponding to a peak THz field strength of a 50 fs THz pulse to >10 MV cm^−1^ if focused to an area of ~(100 μm)^2^.

## Supplementary information

Supplementary Information

## Data Availability

All data are available in the main text or the supplementary materials.
