# Supplementary material for: A novel scheme for ultrashort terahertz pulse generation over a gapless wide spectral range: Raman-resonance-enhanced four-wave mixing
Source: Light Sci Appl. 2023 Feb 2;12:34. doi: 10.1038/s41377-023-01071-z (PMC9894857; doi:10.1038/s41377-023-01071-z)
Supplement: Supplementary file 1 — Supplementary Information [file 41377_2023_1071_MOESM1_ESM.docx]

**Supplementary Information for**

**A Novel Scheme for Ultrashort Terahertz Pulse Generation over a Gapless Wide Spectral Range: Raman-Resonance-Enhanced Four-Wave Mixing**

Jiaming Le^1,†^, Yudan Su^2,†^, Chuanshan Tian^1,3^*, A. H. Kung^1^, Y. Ron Shen^1,2^*

^1^Department of Physics, State Key Laboratory of Surface Physics and Key Laboratory of Micro- and Nano-Photonic Structure (MOE), Fudan University; Shanghai, 200433, China.

^2^Department of Physics, University of California, Berkeley; California, 94720, USA.

^3^Collaborative Innovation Center of Advanced Microstructures; Nanjing, 210093, China.

^†^These authors contributed equally to this work.

*Corresponding authors. Emails: [yrshen@berkeley.edu](mailto:yrshen@berkeley.edu); cstian@fudan.edu.cn

**1. Theory Considerations**

**Coherent Raman excitation of vibration in diamond.** The resonant four-wave mixing (R-FWM) can be visualized as a two-step process: Raman excitation of a vibrational wave, $\vec{Q}\left(\vec{r},t\right)$, followed by a converting pulse that converts $\vec{Q}\left(\vec{r},t\right)$ to a THz pulse. The excitation of $\vec{Q}\left(\vec{r},t\right)$ is described by [1]

$$\left(\frac{\partial}{\partial t}+i\omega_{\text{R}}+\Gamma\right)\vec{Q}\left(\vec{r},t\right)=i\overleftrightarrow{R}:\vec{E}_{1}\left(\vec{r},t\right)\vec{E}_{2}^{*}\left(\vec{r},t\right)$$

which has the solution

$$\vec{Q}\left(\vec{r},t\right)=e^{-i\omega_{\text{R}}t-\Gamma t}\int_{-\infty}^{t} e^{i\omega_{\text{R}}t'+\Gamma t'}i\overleftrightarrow{R}:\vec{E}_{1}\left(\vec{r},t'\right)\vec{E}_{2}^{*}\left(\vec{r},t'\right)\text{ d}t'$$

Here, with $\overleftrightarrow{M}$ denoting the Raman transition moment. For Gaussian $\vec{E}_{1}$ and $\vec{E}_{2}$ pulses of equal pulse width, $\left|E_{i}\left(t\right)\right|\propto\exp\left(-t^{2}/\tau^{2}\right)$, we have

$\vec{Q}\left(t\right)\equiv f\left(t\right)\vec{Q}_{\text{st}}\equiv\tau e^{\Gamma^{2}\tau^{2}/8-\Gamma t}\cdot\mathrm{erfc}\left(\frac{\Gamma\tau^{2}-4t}{2\sqrt{2}\tau}\right)\cdot\vec{Q}_{\text{st}}$

which depends on the peak amplitude $\vec{E}_{\text{1m}}$ and $\vec{E}_{\text{2m}}$, since $\vec{Q}_{\text{st}}\left(\vec{r},t\right)\propto\vec{E}_{\text{1m}}\left(\vec{r},t\right)\vec{E}_{\text{2m}}^{\text{*}}\left(\vec{r},t\right)$. The pulse width determines how far $\vec{Q}\left(\vec{r},t\right)$ at time *t* approaches the steady value $\vec{Q}_{\text{st}}\left(\vec{r}\right)$ as plotted for the case of diamond with $\omega_{\text{R}}/2\pi=40\text{ THz}$ and 1/Γ = 4 ps in Fig. 1b of the main text. Beating of the converting pulse field $\vec{E}_{3}\left(\vec{r},t\right)$ and $\vec{Q}\left(\vec{r},t\right)$ induces a third-order polarization, $\vec{P}^{\left(3\right)}\left(\omega_{\text{S}},\vec{r},t\right)=\overleftrightarrow{R}:\vec{Q}^{*}\left(\vec{r},t\right)\vec{E}_{3}\left(\vec{r},t\right)$ in the medium. If $\vec{E}_{3}\left(\vec{r},t\right)$ is a fs pulse, $\vec{Q}\left(\vec{r},t\right)$ should hardly vary during the pulse duration of $\vec{E}_{3}\left(\vec{r},t\right)$ and simply convert $\vec{E}_{3}\left(t\right)$ into a THz pulse. We can write $\vec{Q}\left(\vec{r},t\right)\equiv f\left(t\right)\vec{Q}_{\text{st}}\left(\vec{r},t\right)$ and then find

$$\begin{matrix} \vec{P}^{\left(3\right)}\left(\omega_{\text{S}},\vec{r},t\right)=\overleftrightarrow{\chi}_{\text{res}}^{\left(3\right)}\left(t\right):\vec{E}_{1\text{m}}^{*}\left(\vec{r}\right)\vec{E}_{2\text{m}}\left(\vec{r}\right)\vec{E}_{3}\left(\vec{r},t\right) \\ \text{with }\overleftrightarrow{\chi}_{\text{res}}^{\left(3\right)}\left(t\right)=f\left(t\right)\left(\overleftrightarrow{\chi}_{\text{res}}^{\left(3\right)}\right)_{\text{st}} \end{matrix}$$

For input polarizations along [110] of diamond, the third-order nonlinear susceptibility in terms of the nonvanishing $\chi^{\left(3\right)}$ elements of diamond has the expression$\chi^{\left(3\right)}=\left(\chi_{xxyy}^{\left(3\right)}+\chi_{xyxy}^{\left(3\right)}\right)/2$. It is seen from Fig. 1b in the main text that for an *E*_1_/*E*_2_ pulse width of 1.3 ps, $\vec{Q}\left(t\right)$ reaches a peak value of ~30% of $\vec{Q}_{\text{st}}$ in diamond, and correspondingly, $\overleftrightarrow{\chi}_{\text{res}}^{\left(3\right)}\left(t\right)\sim0.3\left(\overleftrightarrow{\chi}_{\text{res}}^{\left(3\right)}\right)_{\text{st}}$.

**THz emission from resonant four-wave mixing in diamond.** Knowing $\vec{P}^{\left(3\right)}\left(\omega_{\text{S}},\vec{r},t\right)$ in the depletion-less input limit, we can find the THz output from R-FWM in diamond by solving the wave equation

$$\left[\nabla\times\left(\nabla\times\right)-\frac{\omega_{\text{S}}^{2}}{c^{2}}\varepsilon_{\text{S}}\cdot\right]\vec{E}\left(\omega_{s},\vec{r},t\right)=\frac{4\pi\omega_{\text{S}}^{2}}{c^{2}}\vec{P}^{\left(3\right)}\left(\omega_{s},\vec{r},t\right)$$

or calculating from the basic dipole radiation equation [1]

$$\vec{E}\left(\omega_{\text{S}},\vec{r},t\right)=\left(\frac{\omega_{\text{S}}}{c}\right)^{2}\int_{V} \text{d}^{3}r'\left(1-\hat{r}\hat{r}\right)\cdot\frac{\vec{P}^{\left(3\right)}\left(\omega_{\text{S}},\vec{r},t\right)e^{ik_{\text{S}}\left|\vec{r}-\vec{r}'\right|}}{\left|\vec{r}-\vec{r}'\right|}$$

Phase matching plays an important role.

**Noncollinear phase matching.** With the center frequencies of $\vec{E}_{1}$ and $\vec{E}_{2}$ set at 206 and 166 THz, terahertz generation by R-FWM in diamond can be noncollinearly phase matched (PM). With reference to the center frequency of$\vec{E}_{1}$,$\vec{E}_{2}$ and $\vec{E}_{3}$ in the infinite plane wave approximation, and the beam geometry ($\vec{k}_{2}$||$\vec{k}_{3}$ at an angle *θ* from $\vec{k}_{1}$) sketched in Fig. S1a, the PM angle *θ* as a function of the center THz frequency can be calculated from the dispersion curve of the refractive index of diamond in Fig. S1b and is plotted in Fig. S1c. In the pulse case, we need to consider the finite bandwidths of pulses. The THz output bandwidth is significant if $\vec{E}_{3}$is a fs pulse. In our study with a given *θ*  and $\vec{k}_{2}$||$\vec{k}_{3}$ incident on a diamond plate of *l* = 0.5 mm thick, THz generation with a 60-fs $\vec{E}_{3}$pulse that satisfies the near-PM condition, $\left|\Delta k_{z}\right|l/2=\left|k_{2,z}+k_{3,z}-k_{1,z}-k_{\text{S,}z}\right|l/2\leq1$, and of course also the boundary condition $k_{2,x}+k_{3,x}-k_{1,x}-k_{\text{S},x}=0$ (*z* along the surface normal), should have an appreciable bandwidth and angular spread. An example is presented in Fig. 1d of the main text for the case of *θ* = 0.42°.

To find the angular spread of THz output outside the diamond plate, we must take into account refraction and diffraction of different THz frequency components. The field and energy distributions of each frequency component can then be calculated.

**Plane wave approximation and numerical calculation.** We calculated the THz output from R-FWM in diamond using experimental input parameters. The three input pulses, $\vec{E}_{1}$, $\vec{E}_{2}$ and $\vec{E}_{3}$, could be well approximated by Gaussian both spatially and temporally. They carried energies of 120, 70, and 10 μJ per pulse and focused concentrically and essentially collinearly to π(230×170)/2 μm^2^, π(280×210)/2 μm^2^ and π(230×170)/2 μm^2^, respectively (defined as *A* = *πw_x_w_y_*/2), into the 0.5-mm diamond plate tilted at 45°. The pulse width of$\vec{E}_{1}$and$\vec{E}_{2}$was 1.3 ps (defined as the full width at half maximum) and that of$\vec{E}_{3}$was 63 fs. The three pulses induced a third-order nonlinear polarization $\vec{P}^{\left(3\right)}\left(\vec{r},t\right)$ that had Gaussian spatial and temporal profiles with cross-section of π(140×100)/2 μm^2^ and pulse width of 63 fs resembling that of the $\vec{E}_{3}$ pulse. The explicit expression of $\vec{P}^{\left(3\right)}\left(\vec{r},t\right)$ allowed us to calculate the THz output.

**Plane wave approximation.** We express the three input pulses as $\vec{E}_{i}\left(\omega_{i},\vec{r},t\right)=\vec{E}_{i}\left(\vec{r},t\right)\exp\left(i\vec{k}_{i}\cdot\vec{r}-i\omega_{i}t\right)$, with *i* = 1, 2 and 3 denoting their center frequencies with $\left|\vec{E}_{i}\left(\vec{r},t\right)\right|$ assuming Gaussian profiles. The induced third-order polarization, $\vec{P}^{\left(3\right)}\left(\omega_{\text{S}},\vec{r},t\right)=\vec{P}^{\left(3\right)}\left(\vec{r},t\right)\exp\left[i\left(\vec{k}_{2}+\vec{k}_{3}-\vec{k}_{1}\right)\cdot\vec{r}-i\omega_{\text{S}}t\right]$ with $\omega_{\text{S}}=\omega_{2}+\omega_{3}-\omega_{1}$ and $\vec{P}^{\left(3\right)}\left(\vec{r},t\right)=\overleftrightarrow{\chi}_{\text{res}}^{\left(3\right)}:\vec{E}_{1}^{*}\left(\vec{r},t\right)\vec{E}_{2}\left(\vec{r},t\right)\vec{E}_{3}\left(\vec{r},t\right)$, appears as a tilted cylinder in the diamond plate with an elliptical (near-circular) Gaussian cross-section of π(140×100)/2 μm^2^ parallel to the diamond surfaces. We note that $\vec{P}^{\left(3\right)}\left(\vec{r},t\right)$ has a frequency bandwidth carrying essentially from the bandwidth of the fs$\vec{E}_{3}$pulse; contribution from the bandwidths of $\vec{E}_{1}$ and $\vec{E}_{2}$ are negligible. Each frequency component $\vec{P}^{\left(3\right)}(\vec{r},\omega_{\text{S}}+\nu)$ acts as the source of generating the THz frequency component at $\omega_{s}+\nu$ that emits in directions satisfying the boundary condition for the R-FWM. Under the plane wave approximation, the solution of the wave equation shows that the emitted THz field in diamond at *ω*_S_ +*ν* and $\vec{k}_{\text{S,}\nu}$ is given by

Here,$\vec{r}_{\text{o}}$ refers to the position in the beam overlappipng area at the entrance surface of the diamond plate, and $\Delta k_{\nu,z}=k_{2,z}+k_{3,z}-k_{1,z}-k_{\text{S,}\nu,z}$. Thus, for each THz frequency component, we can find the output energy by integrating $\left|\vec{E}\left(\omega_{\text{S}}+\nu,\vec{r}_{\text{o}}\right)\right|^{2}$ over the beam overlapping area. The THz wave vector $\vec{k}_{\text{S,}\nu}$ is generally tilted away from the input wave vectors $\vec{k}_{1}$ and $\vec{k}_{2}$ so that the THz beam may walk away from the cylinder of $\vec{P}^{\left(3\right)}\left(\vec{r},t\right)$ in diamond. This walk-off effect reducing the THz output becomes more appreciable with decrease of the THz frequency but is negligible above 10 THz. To find THz output from diamond, we need to include change of emitted direction of each THz frequency component due to refraction at the exit surface and the refraction loss. With our beam-sample geometry, frequency components below 2.5 THz are cut off because of total internal refraction in diamond.

**Numerical calculation versus plane wave approximation.** With $\vec{P}^{\left(3\right)}\left(\omega_{\text{S}},\vec{r},t\right)$ known, we can also find THz output directly from the dipole radiation equation by numerical calculation [2]. We can calculate, for a given beam-sample geometry, the spectrum of the total THz output as well as the angular spectral distribution in space. Figure S2 shows the calculated total THz spectra from numerical calculation and plane wave approximation for three representative cases, *θ* between$\vec{k}_{1}$and$\vec{k}_{2}$||$\vec{k}_{3}$at 0.42°, 0.51° and 0.59° for PM THz generation at center frequencies 5, 7.5, and 10 THz, respectively. The agreement between the two approaches is good. For the *θ* = 0.42° case, the calculated spatial distributions for different THz frequency components in the collimated THz output beam are described in Fig. S3. Each frequency component emitted from the beam overlapping area has a near-circular Gaussian energy density distribution in the collimated beam. The circle of the 5 THz component is centered along the surface normal and those of other frequency components shifted from the surface normal along the line defined by the incidence plane. This calculation allows us to map out the spatial variation of the THz output spectrum.

**Comparison of diamond with other centrosymmetric Raman media.** There are other centrosymmetric media with strong Raman resonance, but weak phonon absorption such as crystalline Si, BN, and liquid nitrogen and oxygen. We show in Table S1 comparison of diamond with Si, liquid N_2_ and 30-atm H_2_ gas. Liquid N_2_ and silicon have a larger Raman cross-section or gain than diamond. But liquid N_2_ has a much longer dephasing time, *T*_2_, and a molecular-correlation-induced electric-dipole absorption in the few THz region. It is not known what is the optical damage threshold of liquid N_2_, most likely significantly lower than diamond. As for silicon, the Raman vibrational frequency is much lower than diamond requiring the converting pulse also in the THz range. The optical damage threshold of Si is also significantly lower than diamond. Thus, at this moment, diamond appears to be the best for R-FWM to generate fs THz pulses.

**Thickness dependence of THz output.** The thickness of the diamond is set to 0.5 mm in the above calculations to compare with experiment. For a thicker diamond plate, we expect larger output pulse energy as well as a narrower output spectrum (broader THz pulse). The latter is mainly caused by the beam walk-off effect and the smaller phase-matching acceptance bandwidth in a thicker diamond. In Fig. S4 we compare the simulated THz output spectra from diamond plates with thickness of 0.5 and 1 mm. For the THz output phase-matched at 5 THz (Fig. S4a), the output energy from the 1-mm diamond is 1.9 times larger than the 0.5-mm one, while for output phase-matched at 17 THz, this ratio is 3.2.

**2. Experimental Arrangement**

Our experimental arrangement is sketched in Fig. S5a and the beam geometry on the sample in Fig. S5b. It started from a commercial 33-fs (full width at half maxima) Ti:sapphire oscillator-amplifier system operating at 1 kHz with 5 mJ per pulse output at 800 nm. The system was used to pump two optical parametric amplifiers (OPA) seeded by a common white light source. The signal and idler of OPA1 were 47-fs pulses tuned to *ω*_1_/2*π* = 206 THz and *ω*_2_/2*π* = 166 THz. They were separately stretched, after passing through ZnSe rods of respective path lengths of 50 and 67 mm, into 1.3-ps positively frequency-chirped pulses that had a constant instantaneous beat frequency with a narrow band width (Fig. S5c). The two pulses of energies 120 μJ and 70 μJ with Gaussian spatial and temporal profiles, were focused to areas of π(260×160 μm^2^)/2 and π(300×200 μm^2^)/2, respectively, into a 500-μm (001)-cut CVD diamond plate, tilted at 45° from the beams, to excite the Raman resonance at 40 THz. (The beam profile was measured by the knife-edge cutting scheme and found to be well described by an intensity distribution $I=I_{\text{m}}\exp\left(-2x^{2}/w_{x}^{2}\right)\exp\left(-2y^{2}/w_{y}^{2}\right)$, with the beam area defined by *A* = *πw_x_w_y_*/2.) A third beam with tunable *ω*_3_/2*π* from 45 to 60 THz and with also a Gaussian spatial and temporal profile was a 63-fs pulse derived from a difference frequency generation stage pumped by the signal and idler of OPA2. It was focused to overlap with the *ω*_1_ and *ω*_2_ beams in an area of π(180×140 μm^2^)/2 inside diamond at a selected time when the vibrational excitation was close to the peak (Fig. 1b of the main text). (The optical damage thresholds for our diamond plate were 0.4 TW cm^-2^ for the 1.3-ps *ω*_1_ and *ω*_2_ pulses and 7 TW cm^-2^ for the 63 fs *ω*_3_ pulse.) The THz output from diamond was characterized spatially, spectrally and temporally. The THz beam from the diamond plate was refocused to an image plane by two parabolic mirrors, and a knife edge moving in the image plane was used to measure the spatial profile of the total THz output at the exit surface of the diamond (Fig. S5a(A)). With the knife edge removed, the pyroelectric detector measured the total THz output energy. The angularly spread THz output was collimated by a parabolic mirror to a ~0.5 cm beam and directed into a Fourier transform infrared spectrometer (FTIR) to obtain its spectrum (Fig. S5a(B)). A movable slit or aperture was inserted before the FTIR to learn how the THz spectral content varied with angular position. Finally, electro-optic sampling in a 0.1-mm GaP plate was used to measure temporal profile of the THz output pulses (Fig. S5a(C)).

**3. Experimental characterization of THz output**

**THz Beam profile.** The knife-edge cutting scheme was employed to measure the THz beam profile, as described in Fig. S5a(A). THz output emitted from the spot on the exit surface of diamond was focused to an image plane and the knife edge moving in the plane cut into the beam to measure the spatial beam profile. The result showed a signal change well described by an erf function, indicating that the spatial profile of the THz field at the diamond surface was nearly Gaussian as expected. With 1:1 magnification ratio of imaging, the THz spot size at focus was measured to be π(190×120)/2 μm^2^.

**Spectroscopic measurements.** A home-made FTIR with an aperture of 50×35 mm^2^ coupled with a pyroelectric detector was used for THz spectroscopy measurements. A 1-mm high-resistivity Si plate was inserted in the beam before the FTIR so that the total THz output energy could be concurrently measured by a separate pyroelectric detector. The results are presented in Fig. 2a of the main text.

To map out the spatial variation of the THz output spectrum, we place a slit with variable slit width at different positions perpendicular to the incidence plane in the collimated beam. THz light leaking through the slit was measured by the FTIR. Fig. S6a and S6b give, respectively, the measured spectra for a 1-mm slit centered on the circles of 5, 7, and 9 THz and for a 0.5-mm slit centered on the circles of 13, 15, and 17 THz. The numerically calculated spectra (solid curves) fit well with the experiment. For slits of widths of 0.5, 1, and 2 mm centered at the circle of 15 THz, the measured and calculated THz spectra are plotted in Fig. S6c. The spectrum is more intense with a wider slit as it should but shows not much change in width.

For material studies, well-defined transform-limited fs THz pulses are preferred. As shown in Fig. S7, the THz output from diamond can be collimated by a parabolic mirror (OAP 1) with its different frequency components spatially separated but propagating in the same direction. If the collimated beam is refocused by another parabolic mirror (OAP 2) to an image spot, the different frequency components are recombined in space and time to form a near-transform-limited THz pulse. This was confirmed by the measured spectral distribution in the THz beam described above and the temporal profile of the THz pulse at the image spot together with its flat spectral phase shown in Fig. 3(c) and 3(d), respectively, in the main text.

**Temporal measurements of THz pulses.** To characterize the THz output in the time domain, electro-optic sampling in a 100-μm GaP(110) plate was used to measure the time variation of THz pulses at the image spot. We take the case of THz pulses generated from diamond with noncollinear phase matching set at 5 THz as an example. The temporal trace of the THz pulse with each data point obtained from accumulation of 100 shots, is displayed in Fig. 3c, and its Fourier-transform and phase spectra in Fig. 3d of the main text. The trace was distorted by absorption at higher THz frequencies in GaP, resulting in a broader pulse and a corresponding narrower spectrum compared to that measured directly by FTIR.

**Stability and tuning of carrier-envelop phase.** In our experiment, because the signal pulses of the two OPAs were seeded by the same white light source, their carrier-envelop phases (CEP) were locked to the CEP of the pump laser. The idler pulses from the OPAs should have a stable CEP even if the pump CEP varied from shot to shot [3]. The CEP of the *ω*_3_ pulse from difference frequency generation between the signal and the idler of OPA-2 was also locked to the pump. We then expected that the CEP of the THz output pulse from four-wave mixing of *ω*_s_ =*ω*_3_ – *ω*_1_ + *ω*_2_ should be stable despite the shot-to-shot CEP variation of the pump. This is evidenced by the clear temporal trace of the THz pulse in Fig. 3c of the main text considering that each data point was obtained from accumulation of signals over 100 shots in the measurement. The CEP of the THz pulse could be varied by varying the time of the *ω*_3_ pulse arriving at the diamond plate. Fig. 3e of the main text shows two measured THz pulses with CEP adjusted to 0 and π; it was a time delay of 13 fs of the *ω*_3_ pulse that led to a CEP change of π.

**References**

[1] Shen, Y. R. Stimulated Raman scattering. in *The Principles of Nonlinear Optics* (J. Wiley, 1984), Chapter 10.

[2] Morris, J. R. & Shen, Y. R. Theory of far-infrared generation by optical mixing. *Phys. Rev. A* **15**, 1143–1156 (1977).

[3] Rossi, G. M. *et al.* CEP dependence of signal and idler upon pump-seed synchronization in optical parametric amplifiers. *Opt. Lett.* **43**, 178-181 (2018).

[4] Hanna, D., Pointer, D., & Pratt, D. Stimulated Raman scattering of picosecond light pulses in hydrogen, deuterium, and methane. *IEEE Journal of Quantum Electronic*s **22**, 332-336 (1986).

[5] Grun, J. B., McQuillan, A. K., & Stoicheff, B. P. Intensity and gain measurements on the stimulated Raman emission in liquid O_2_ and N_2_. *Physical Review* **180**, 61 (1969).

[6] Claps, R. *et al*. Observation of stimulated Raman scattering in silicon waveguides. *Optics Express* **11**, 1731–1739 (2003).

[7] Savitski, V. G., Reilly, S., & Kemp, A. J. Steady-state Raman gain in diamond as a function of pump wavelength. *IEEE Journal of Quantum Electronics* **49**, 218-223 (2013).


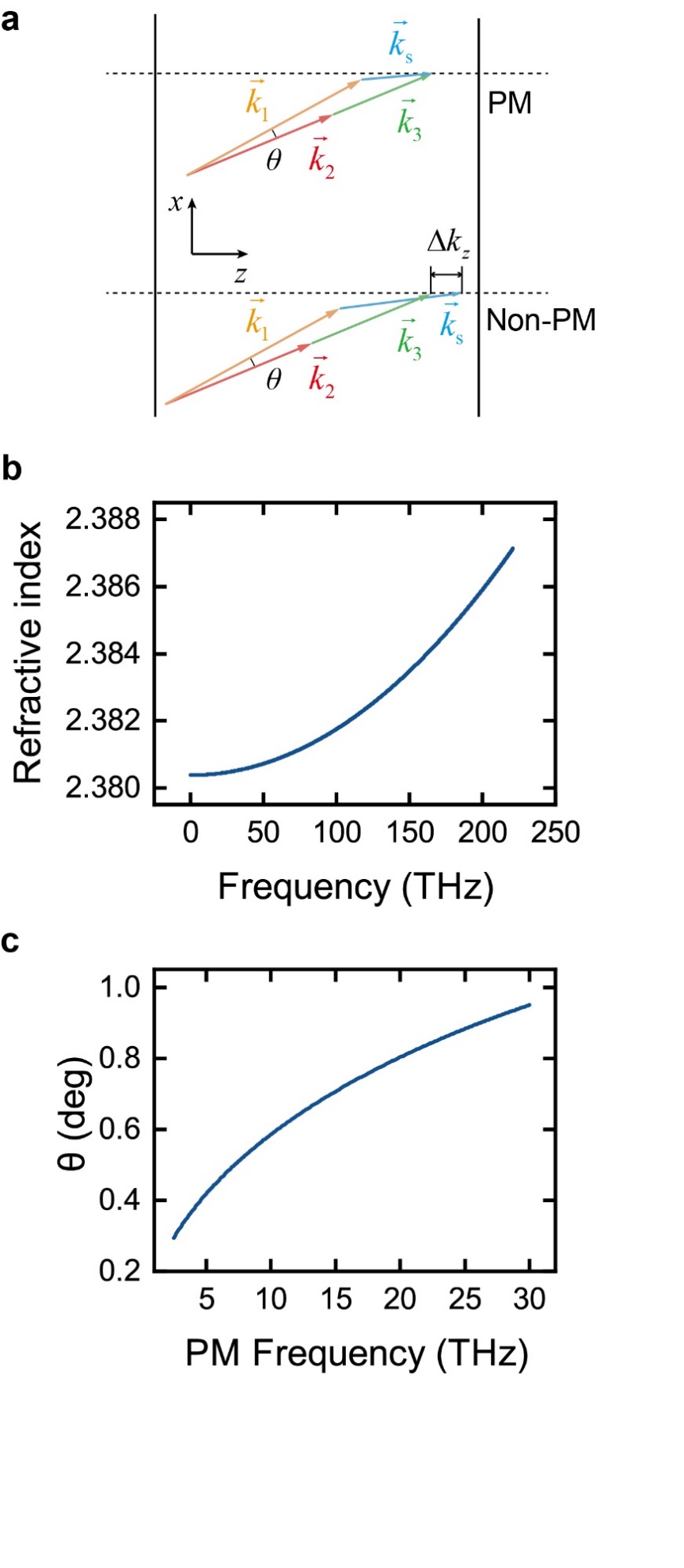


**Fig. S1 | Noncollinear phase matching.** **a,** Sketch of beam geometry. **b,** Dispersion of refractive index of diamond. **c,** PM angle *θ* as function of PM THz frequency.


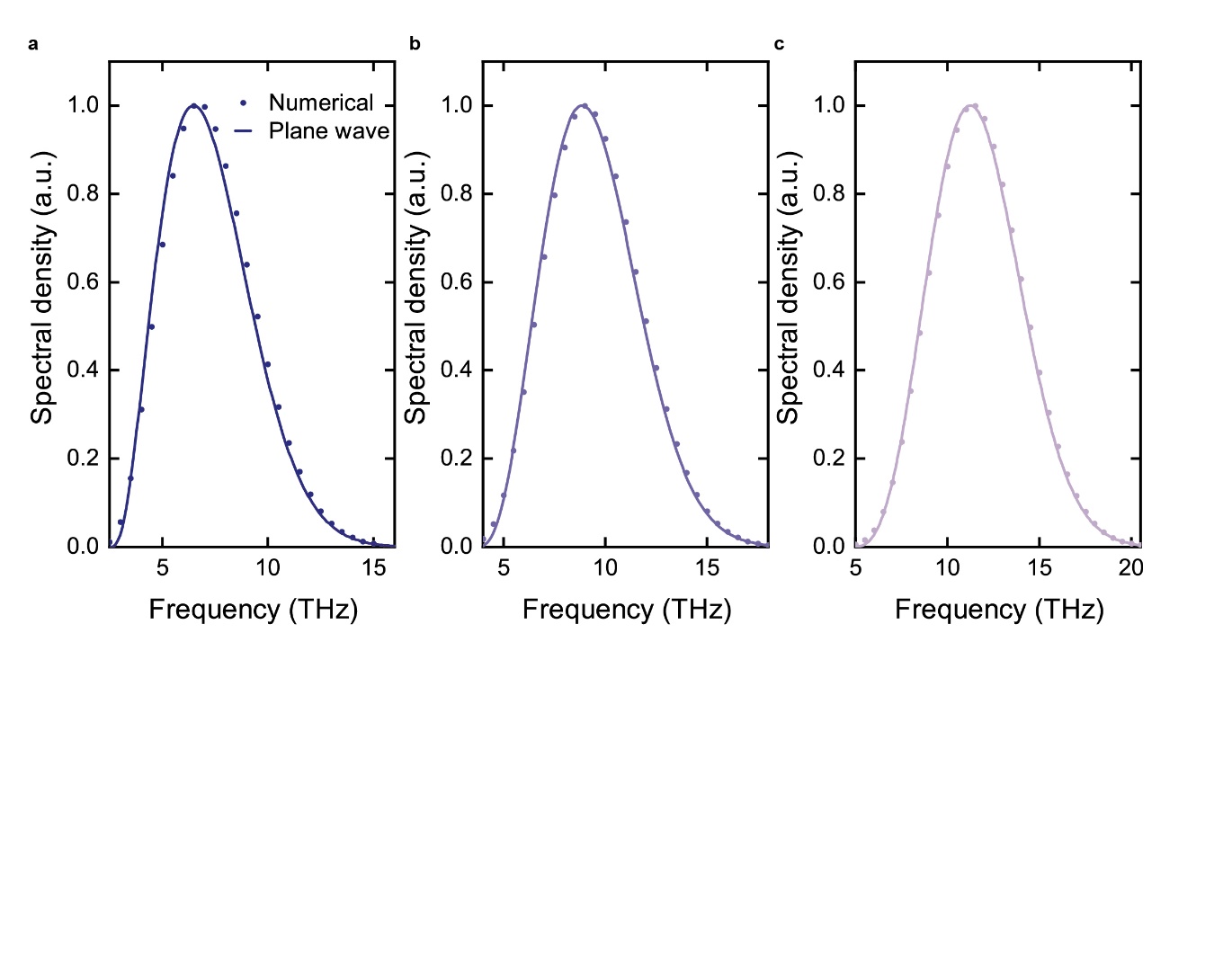
**Fig. S2 | Comparison between numerical calculation and plane wave approximation.** Calculated total THz output spectra from numerical calculation (dots) and plane wave approximation (curves). The phase matching angles are 0.42 deg in (a), 0.51 deg in (b) and 0.59 deg in (c).


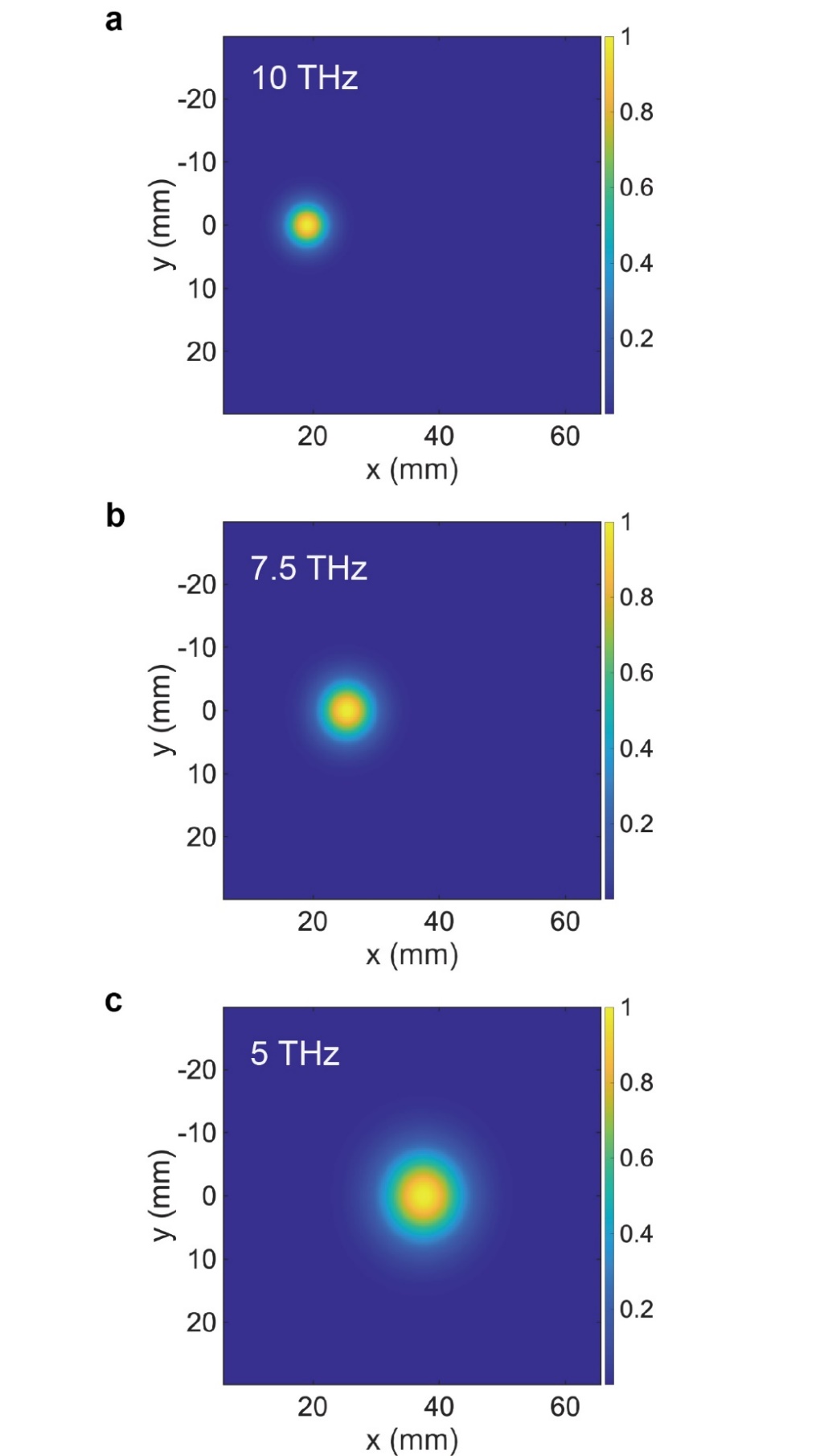


**Fig. S3 |** Spatial energy distributions of different THz frequency components in a collimated beam from numerical calculation.


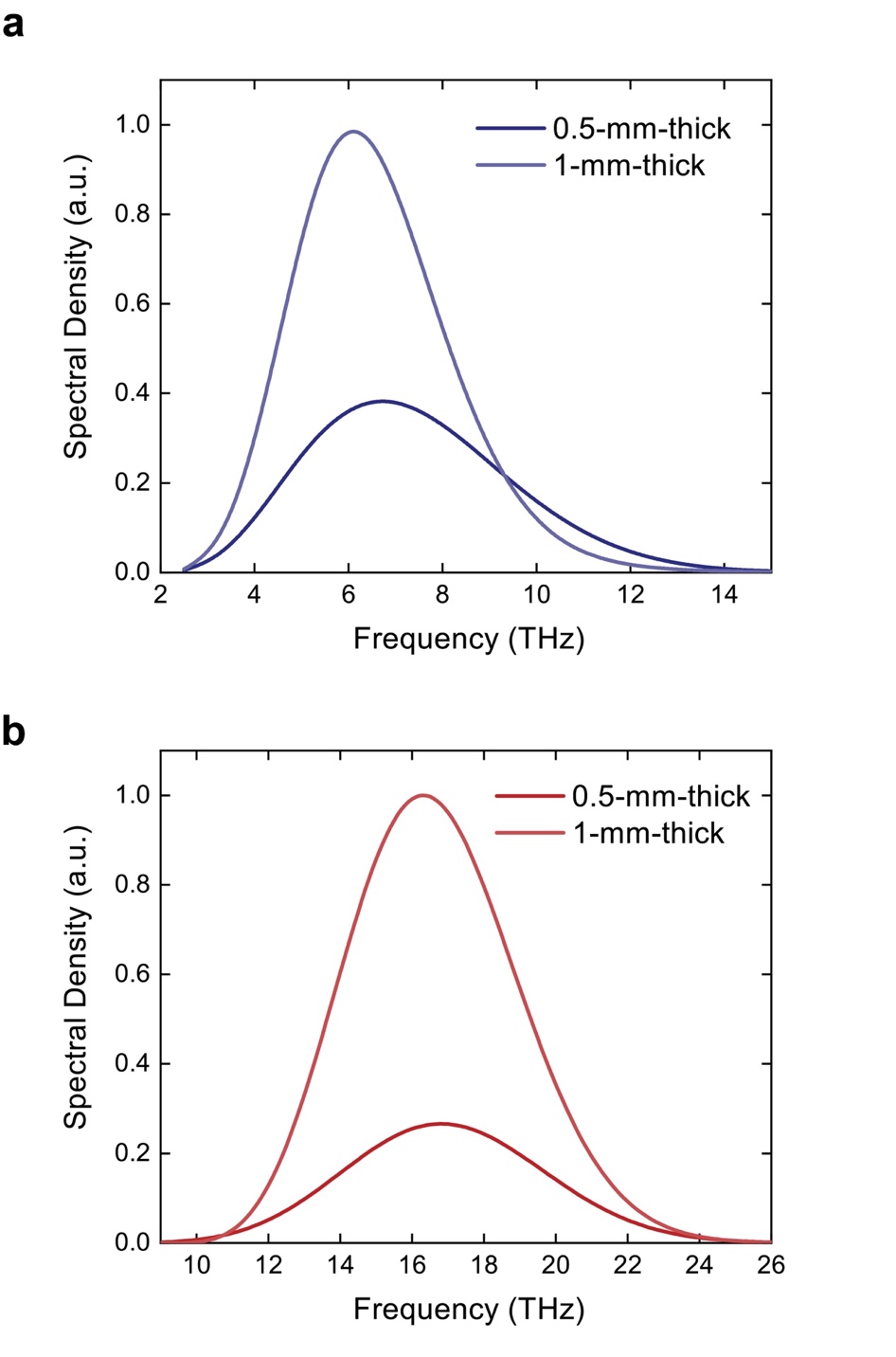


**Fig. S4 |** Simulated THz output spectra from diamond with two different thicknesses. **a,** phase-matched at 5 THz. **b,** phase-matched at 17 THz.

**
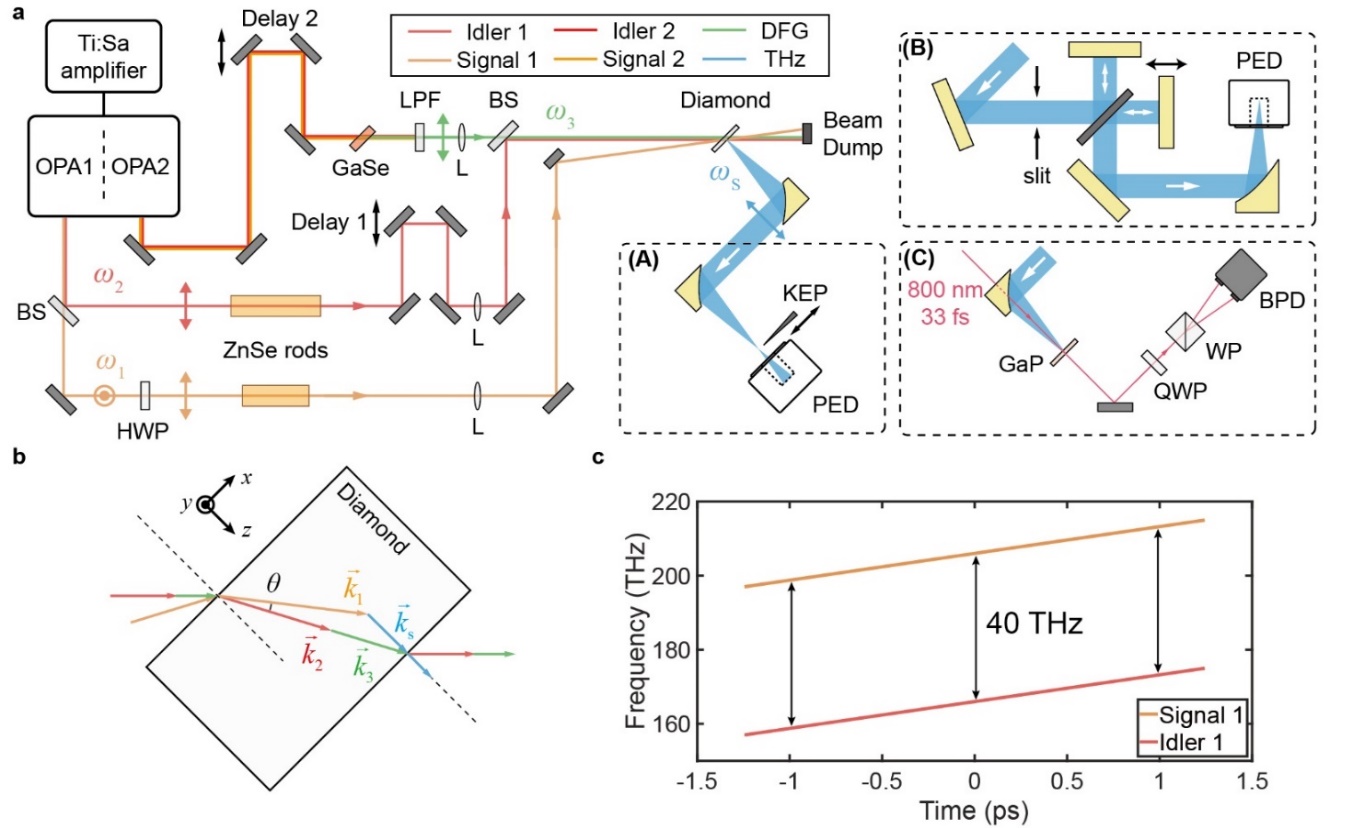
**

**Fig. S5 | Experimental arrangement for resonant four-wave mixing in diamond. a**, Experimental setup: BS, dichroic beam splitter; HWP, half wave plate; LPF, low pass filter; L, lens; PED, pyroelectric detector; KEP, knife edge plate; QWP, quarter wave plate; WP, Wollaston prism; BPD, balanced photo-detector. The output THz pulse was characterized by (A) knife edge cutting into the beam in the image plane to measure the spatial profile of the energy distribution; (B) FTIR spectrometer to measure the spectrum; (C) electro-optic sampling to measure the temporal profile. **b,** Sketch of beam geometry with respect to the diamond plate. **c**, Stretched and positively chirped *E*_1_ and *E*_2_ pulses with a constant difference frequency in time.


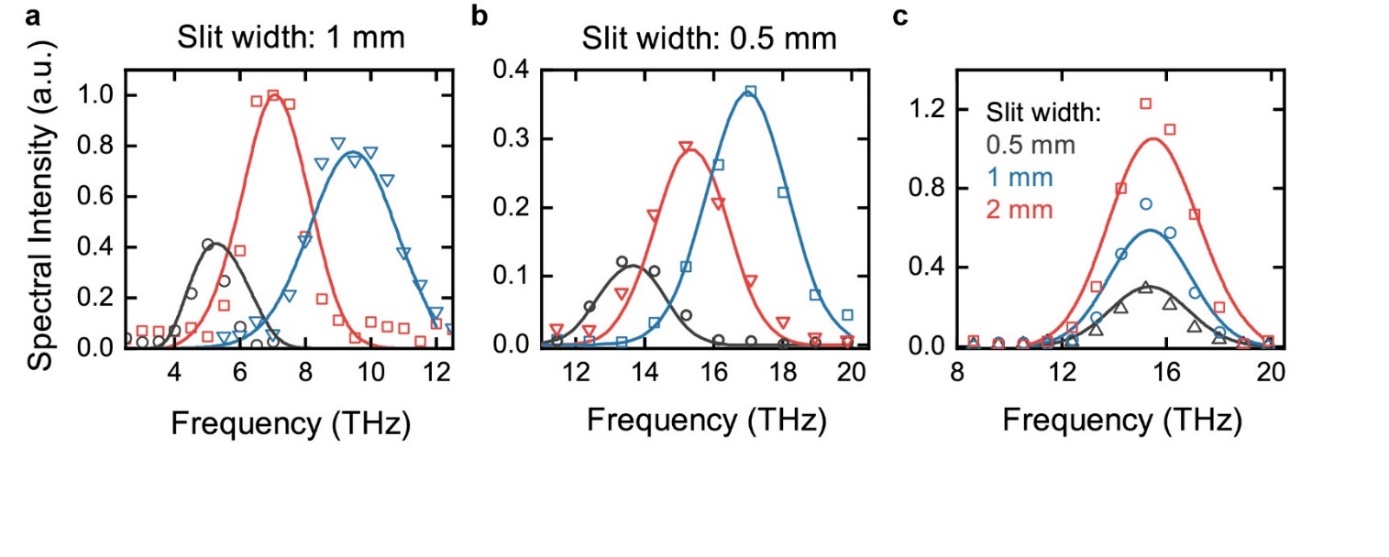


**Fig. S6 | Spectra of THz output passing through a slit of given width at a given position in the collimated THz output beam:** **a**, 1-mm slit at the centers of the circles for 5 THz (black circles), 7 THz (red squares), and 9 THz (blue triangles). **b**, 0.5-mm slit at the centers of the circles for 11 THz (black circles), 13 THz (red triangles), and 15 THz (blue squares). **c**, Slits of 0.5 mm (black triangles), 1 mm (blue circles) and 2 mm (red squares) at the center of the circle for 15 THz in (b). Solid curves are theoretical predictions.


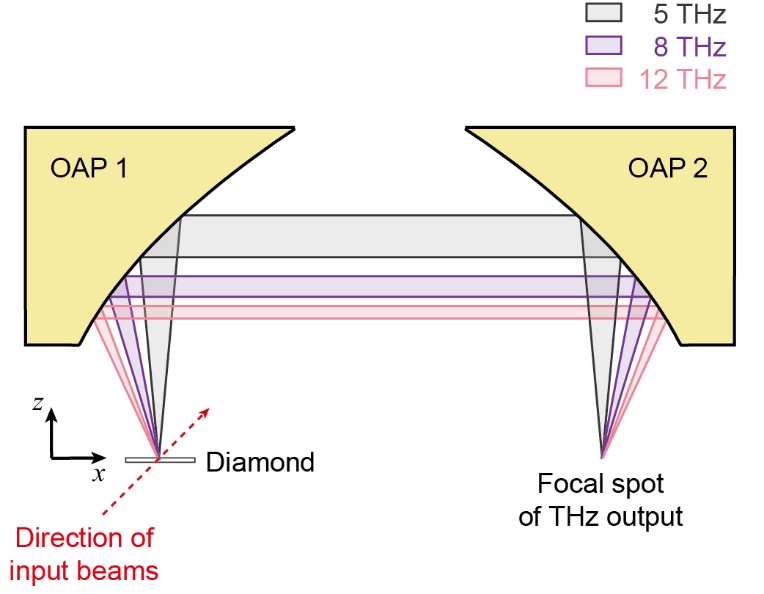


**Fig. S7 | Schematic of collimation and re-focusing of spatially chirped THz output.**

**Table S1 | Comparison of centrosymmetric Raman media**

| Medium | Raman vibration frequency | Dephasing time | Steady state Raman gain | Reference |
| --- | --- | --- | --- | --- |
| Gaseous H_2_ (30 atm) | 124.6 THz | 208 ps | 0.9 cm GW^-1^  (*λ*_pump_ = 1.06 μm) | Ref. [4] |
| Liquid N_2_ | 69.7 THz | 80 ps**^a^** | 16 cm GW^-1^  (*λ*_pump_ = 0.69 μm) | Ref. [5] |
| Silicon | 15.6 THz | 1.3 ps**^a^** | 20 cm GW^-1^  (*λ*_pump_ = 1.55 μm) | Ref. [6] |
| Diamond | 40.0 THz | 3.7 ps | 10 cm GW^-1^  (*λ*_pump_ = 1.2 μm) | Ref. [7] |

**^a^** The dephasing time is converted from the Raman linewidth.
